# Mechanism of Hedyotis Diffusa in the Treatment of Cervical Cancer

**DOI:** 10.3389/fphar.2021.808144

**Published:** 2021-12-15

**Authors:** Kai Qian, Dan Fu, Baorui Jiang, Yue Wang, Fei Tian, Li Song, Lei Li

**Affiliations:** ^1^ Department of Cardiology, Institute of Cardiovascular Development and Translational Medicine, The Second Affiliated Hospital of Wenzhou Medical University, Wenzhou, China; ^2^ Medical College of YiChun University, Yichun, China

**Keywords:** hedyotis diffusa, component, cervical cancer, mechanism, network pharmacology

## Abstract

Cervical cancer is one of the most common malignant tumors among women in the world. In clinical practice, Hedyotis diffusa has pharmacological effects in treating cervical cancer, but its components are relatively complex, and the mechanism of Hedyotis diffusa in treating cervical cancer is still unclear. In this work, the potential active components and mechanism of Hedyotis diffusa in the treatment of cervical cancer were explored by means of network pharmacology. By constructing its active ingredient-target network, and enriching and analyzing the targets, we found the key targets and their effective components (beta-Sitosterol and Quercetin) that play a therapeutic role. Finally, we evaluated the prognostic value of the core target genes through survival analysis. Our work initially explored the therapeutic mechanism of cervical cancer, which lays a theoretical foundation for further exploring its pharmacological action and its clinical application.

## Introduction

Cervical cancer is the fourth most common female malignant tumor and the most common cause of cancer death in the world (Bray et al., 2018). About half a million women develop cervical cancer every year and about 300,000 people die from the disease every year (Cohen et al., 2019). The incidence and mortality rates of cervical cancer in developed countries are much lower than those in developing countries, which is mainly due to the lack of screening and prevention programs for cervical cancer in many developing countries (Denny, 2012). Cervical cancer is considered as the only one which is known cause and can be preventable among human cancers. Although we know that the high-risk subtype of human papillomavirus (HPV) is the main cause of cervical cancer, the incidence and mortality of cervical cancer have not been significantly reduced (Walboomers et al., 1999). Cervical cancer patients are mainly treated by surgery, radiotherapy and chemotherapy. And novel immunotherapy, such as antibody targeted therapy and adoptive cell therapy, has unsatisfactory efficacy (Dyer et al., 2019). Surgical resection of tumors is mainly applied to early-stage patients, but most patients diagnosed with cervical cancer are generally advanced and lose the best treatment time, so chemotherapy and radiotherapy are the main treatment methods (Li et al., 2016). However, most chemotherapeutic drugs have severe toxic and side effects and long-term use will result in drug resistance, which seriously affect the efficacy. By contrast, Chinese medicinal active ingredients have the advantages of multiple pathways, multiple targets and small adverse reactions in the anti-tumor aspect, and may be more suitable for clinical treatment of cancer patients (Nie et al., 2016). Hedyotis diffusa is panicum miliaceum of Rubiaceae, widely distributed in subtropical regions (Li et al., 2013), and it is a famous traditional Chinese medicine for the treatment of inflammation-related diseases such as hepatitis and appendicitis in China (Chen et al., 2016). Herba hedyotidis diffusae has various pharmacological activity, such as anticancer activity (Liu et al., 2010), antioxidant activity (Lu et al., 2000),immunomodulatory effect (Lin CC. et al., 2011) and anti-inflammatory activity (Ye et al., 2015),etc. At present, more and more clinical researches prove that the oldenlandia diffusa has remarkable anticancer activity (Gupta et al., 2004). Qiao Yan Cai et al. studied the molecular mechanism of Hedyotis diffusa in the treatment of colon cancer, and the results showed that Hedyotis diffusa exerts the curative effect mainly by regulating the expression of several important genes in the Signal Transducer and Activator of Transcription 3 signaling pathway. Hedyotis diffusa can inhibit the phosphorylation of Signal Transducer and Activator of Prescription 3, thereby promoting the apoptosis of tumor cells and inhibiting the proliferation of tumor cells to achieve the curative effect of treating colon cancer (Cai et al., 2012). Studies have shown that one of the mechanisms of Hedyotis diffusa’s participation in tumor treatment is to inhibit tumor angiogenesis (Lin J. et al., 2011). As for the mechanism of treating cervical cancer,proto-oncogene c-Sre is a member of Sre-familykinases, which is a kind of non-receptor protein tyrosine kinase and plays an important role in regulating cell growth, development, differentiation and other biological functions. Studies have shown that proto-oncogene c-Sre can induce malignant transformation of cells. Studies have found that down-regulating the activity of c-Sre kinase can effectively inhibit the proliferation of cervical cancer cells (Kong et al., 2011). However, due to the complex components of Hedyotis diffusa, the mechanism for its treatment of most cancers including cervical cancer is not clear. Network pharmacology was first proposed by the British pharmacologist Hopkins in 2007. It is a new subject based on system biology theory and multi-directional pharmacology theory, which integrates information network science and computer science. And it has the overall and systematic research characteristics (Hopkins, 2008). Network pharmacology can be used to analyze biological system networks, predict and infer the correlation between targets and networks, and then construct a “drug-target-disease” interaction network, which can comprehensively and systematically clarify the intervention and influence of drugs on diseases (Zhang et al., 2014). In this study, with the help of network pharmacology, the effective active compounds of Hedyotis diffusa for treating cervical cancer were systematically screened out, and the pharmacological effects of Hedyotis diffusa for treating cervical cancer with multiple targets, multiple pathways and multiple mechanisms were preliminarily revealed.

## Methods

### Collection of Effective Components and Targets

In TCMSP (http://tcmspw.com/tcmsp.php) database, the chemical components of Herba Hedyotis were collected by retrieving the key word “Herba Hedyotis” and taking oral bioavailability greater than or equal to 30% and drug-like property of 0.18 as screening conditions, the ineffective components were removed. Finally, target information corresponding to the active components is collected through HERB (http://herb.ac.cn/) database and the network of component targets is visualized by Cytoscape.

### Acquisition of Cervical Cancer-Related Targets

By searching the keyword “cervical cancer” in GeneCards database (https://www.genecards.org/), we collected 7,110 related targets. Finally, we used Venn diagram to display the disease targets and drug component action targets, and got 175 common targets.

### Functional Enrichment Analysis

Functional enrichment analysis included the GO functional enrichment analysis and KEGG pathway analysis. The GO functional enrichment analysis mainly included three parts: biological process (BP), molecular function (MF), cellular component (CC). Pathway enrichment analysis and GO enrichment analysis were performed using DAVID database to obtain the results of the top 20 FDR rankings, respectively, and the enrichment results were visualized using BioFormatics (www.bioinformatics.com.cn).

### Construction of Protein Interaction Network and Extraction of Core Targets

Import the intersection targets into STRING database and build PPI network. Then, using cytoHubba in Cytoscape to extract the top ten targets in PPI network by MCC algorithm, and then performing pathway enrichment analysis on the extracted core targets.

### Prognostic Information of Core Targets

KaplanMeie-plotter is a popular website tool based on EGA, TCGA and GEO databases, which is used to evaluate the survival of gene pairs. The prognosis of 10 core targets in cervical cancer was obtained by GEPIA2 (gepia2. cancer-pku.cn), and the targets with *p* value less than or equal to 0.05 were selected.

## Results

### Related Targets of Hedyotis Diffusa in the Treatment of Cervical Cancer

**FIGURE 1:**
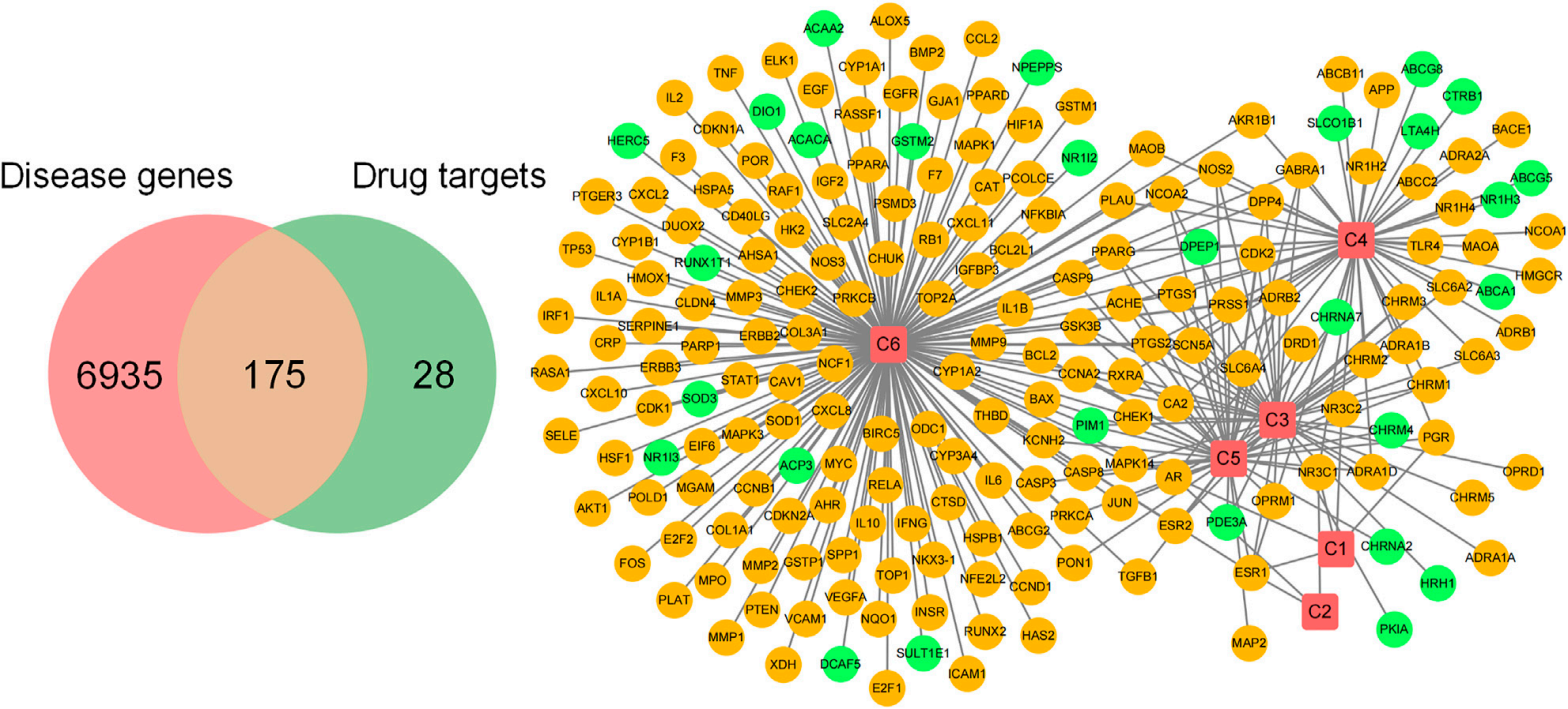
Screening of therapeutic targets and construction of its component-target network.

We screened 7,110 cervical cancer-related targets from Gene Cards database. Six components of Hedyotis diffusa and its 203 action targets were collected through TCMSP database and HERB database, among which there were 175 cervical cancer-related targets (Figure 1). Here, we show the interaction between components and targets in the form of network, as shown in Figure 1. Red squares represent drug components, and circles represent component action targets, among which orange targets represent cervical cancer-related targets and green circles represent non-cervical cancer-related targets. It can be seen that most of the targets in the whole target network are related to cervical cancer, suggesting that Hedyotis diffusa has certain pertinence in the treatment of cervical cancer.

### Functional Enrichment Analysis of Therapeutic Targets for Cervical Cancer

**FIGURE 2:**
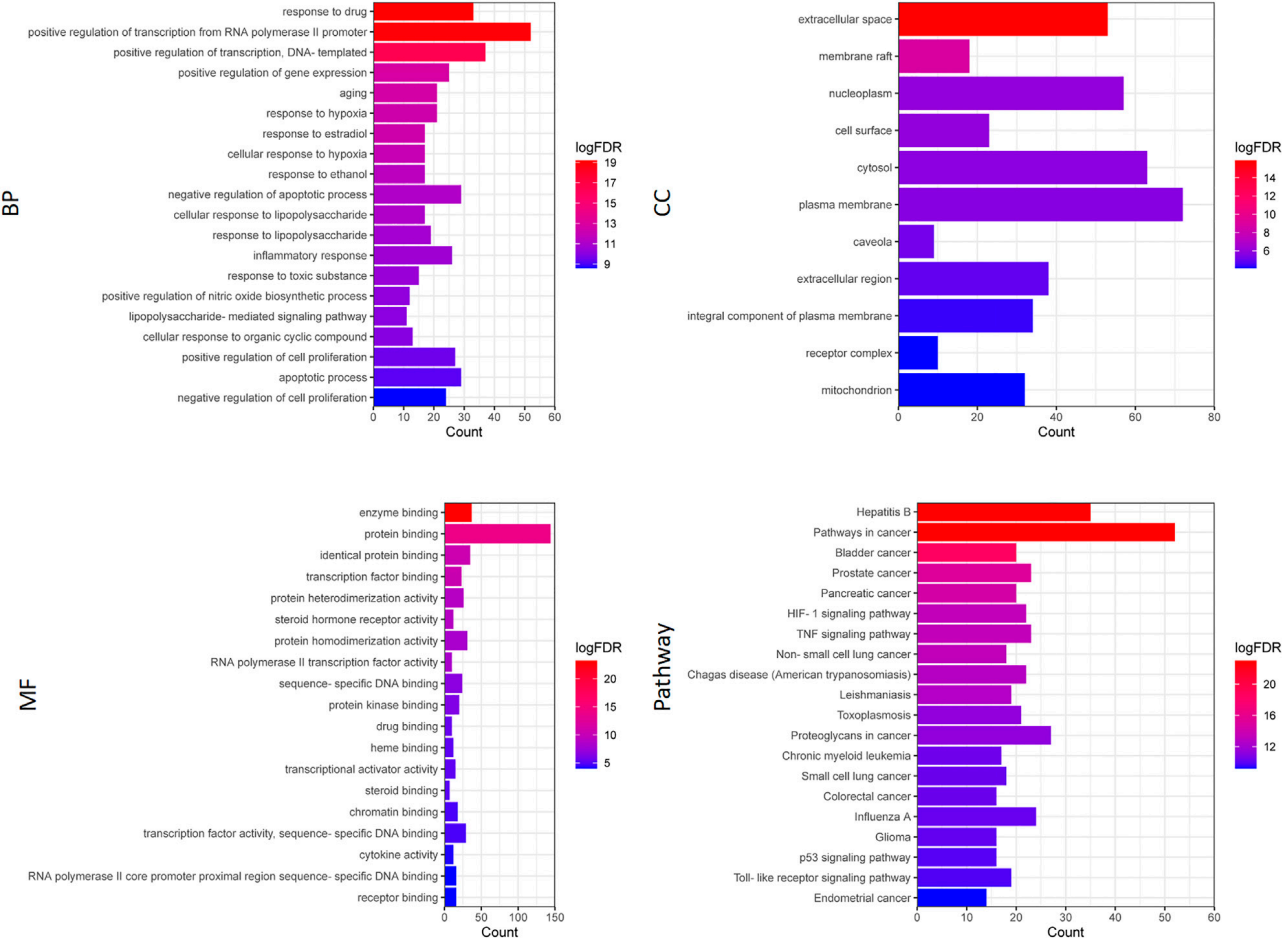
KEGG pathway analysis and GO enrichment analysis.

GO function enrichment and KEGG pathway enrichment of the above 175 common targets were analyzed by DAVID database (https://david.ncifcrf.gov/home.jsp) (Figure 2). GO analysis includes biological processes (BP), cellular components (CC) and molecular functions (MF), which together describe the functions of gene products.

As shown in Figure 2, the pathway enrichment analysis of Hedyotis diffusa in treating cervical cancer mainly involves Pathways in cancer, Hepatitis B, Bladder cancer, Prostate cancer, Pancreatic cancer, TNF signaling pathway, HIF-1 signaling pathway, Non-small cell lung cancer, Chagas disease (American trypanosomiasis), Leishmaniasis and so on. GO analysis mainly involves response to drug, positive regulation of transcription from RNA polymerase II promoter, positive regulation of transcription, DNA-templated, positive regulation of gene expression, extracellular space, membrane raft, cell surface, nucleoplasm, enzyme binding, protein binding, transcription factor binding, etc.

Enrichment analysis showed that Hedyotis diffusa may act on multiple targets through various signal pathways and play a role in the treatment of cervical cancer. At the same time, it also provides reference value for further searching for key core targets and compounds.

### Construction of Protein-Protein Interaction Network and Analysis of its Core Targets

**FIGURE 3:**
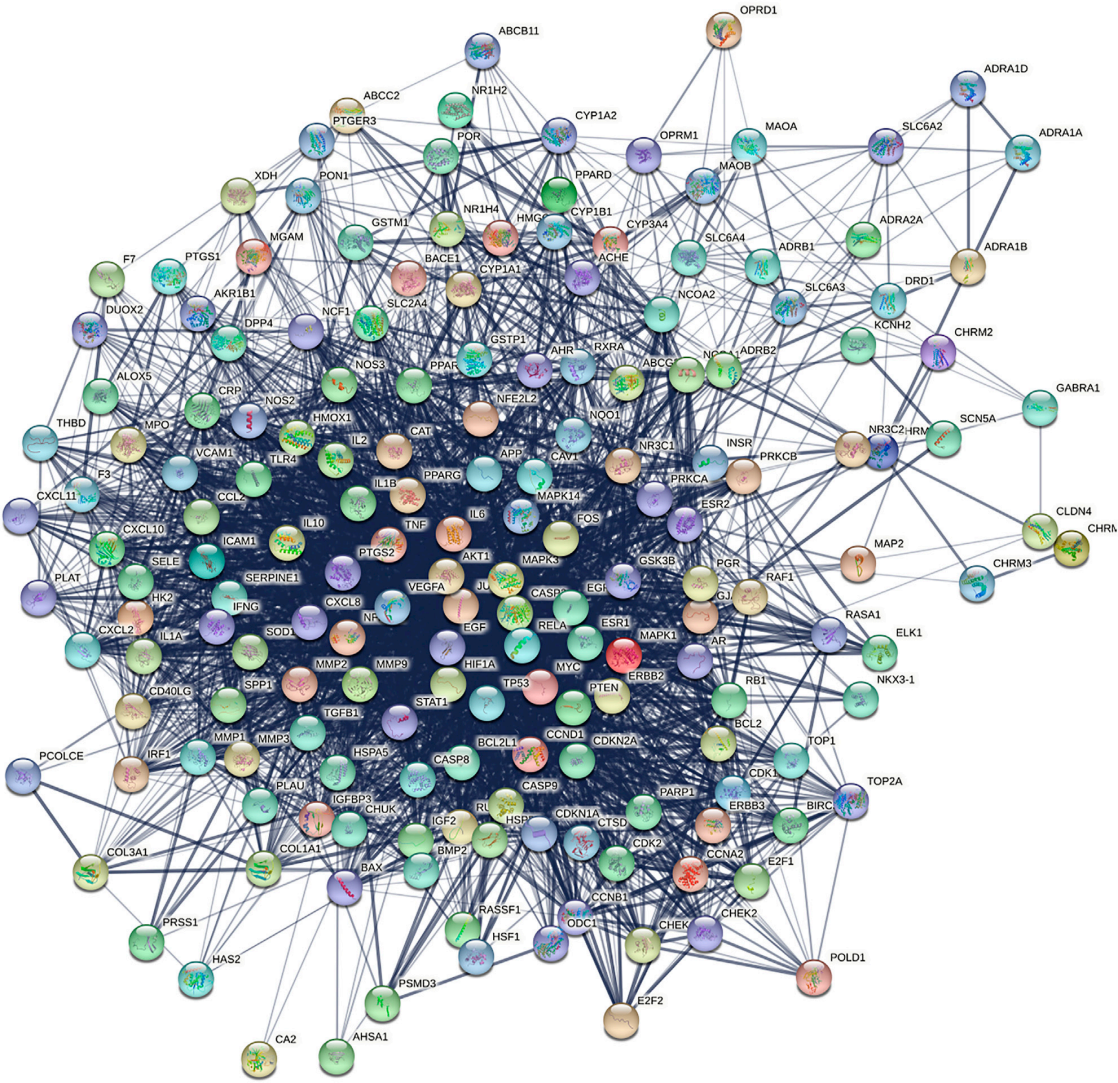
Protein-protein interaction network.

We introduced the screened 175 common target genes into STRING, and set the minimum confidence level to 0.4. PPI network was constructed by the algorithm of STRING database, and the relationship between protein was visualized in the form of network. As shown in Figure 3, the network graph has 175 nodes and 3,424 edges, and the average node degree is 39.1, and the *p* value is less than 1.0e-16. Nodes represent target proteins, and each line represents the interaction between target proteins. The thickness of the connecting line represents.

**FIGURE 4:**
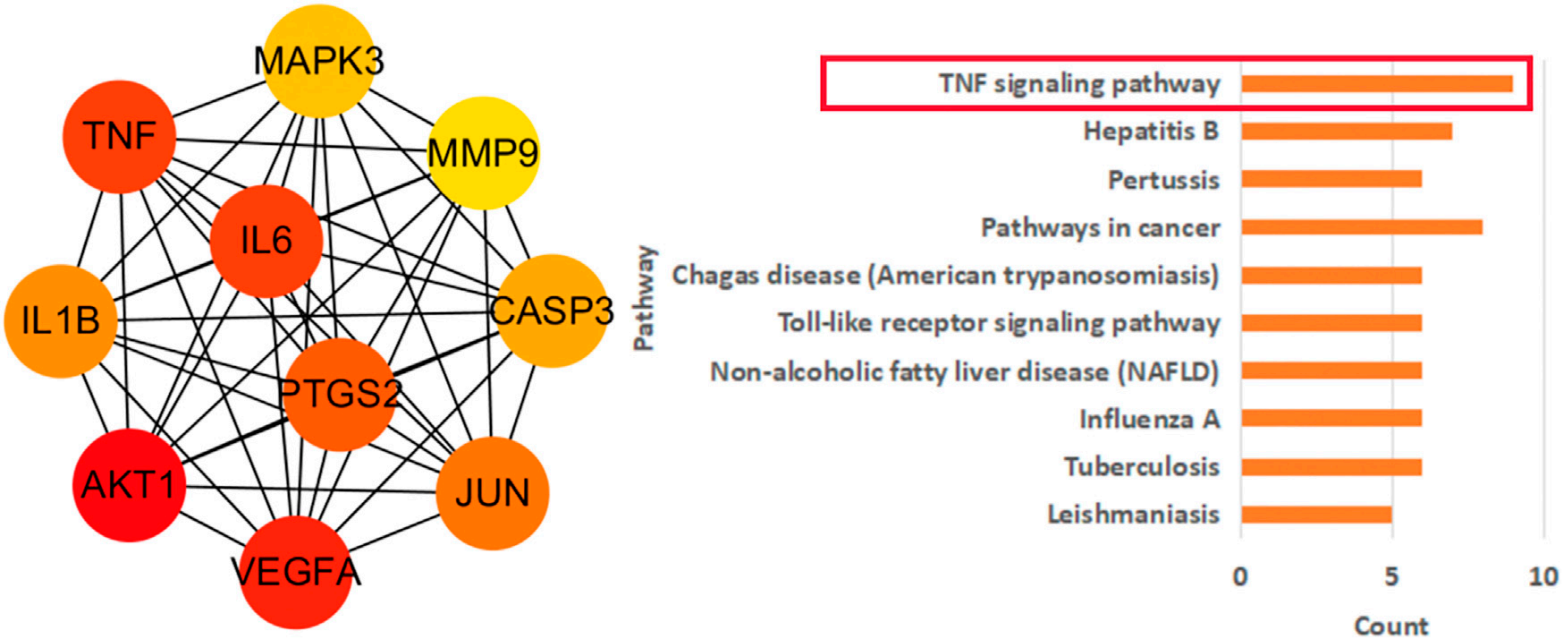
Core target network and its pathway analysis.

In addition, cytoHubba was used for topology analysis of PPI networks, and the top ten core targets were extracted, which were successively AKT1, VEGFA, TNF, IL6, PTGS2, JUN, IL1B, CASP3, MAPK3, and MMP9(Figure 4). Later, we further enriched the pathways for the core targets, with the following results: TNF signaling pathway, Hepatitis B, Pertussis, Pathways in cancer, Chagas disease (American trypanosomiasis), etc. We found that the first-ranked pathways not only had the most significant statistical significance, but also involved more core targets. In addition, this pathway also appeared in our previous enrichment results of the entire PPI network, and it was ranked high, suggesting that Hedyotis diffusa may play an important role by mediating multiple pathways in the treatment of cervical cancer, and TNF signaling pathway may play a key role in the entire treatment network.

### Prognostic Analysis of Core Targets and Construction of Component Target Network

**FIGURE 5:**
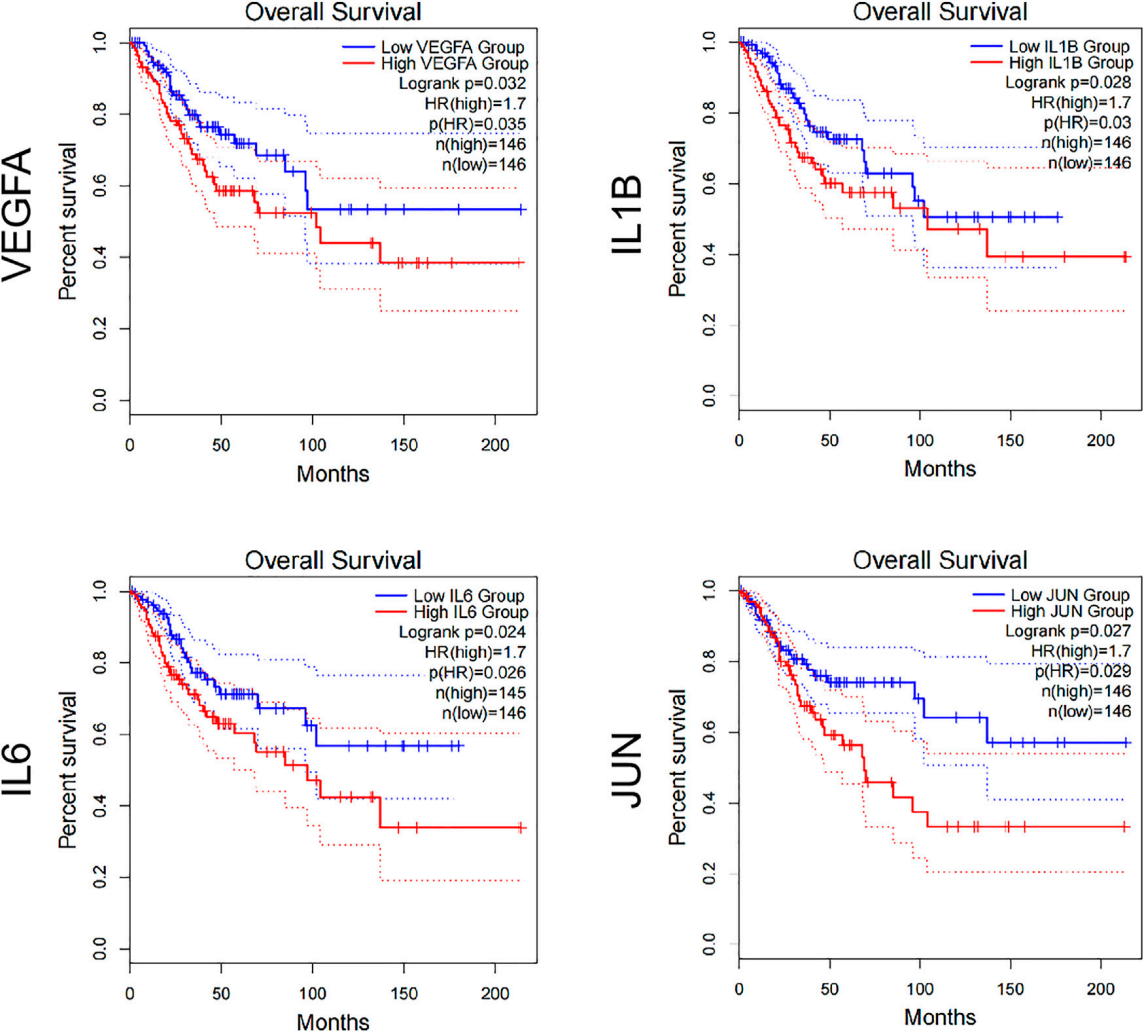
Overall survival of core target.

In order to further determine the role of the core target in the network, we analyzed the overall survival of the core target by using GEPIA2. As shown in Figure 5, the results show that four of the 10 core targets have prognostic value in cervical cancer, namely VEGFA, IL1B, IL6, and JUN. The prognosis of all these targets is poor (HR = 1.7), and the low expression in cervical cancer has a good overall survival time. It also suggests that the components interacting with these targets may play the role of small molecule inhibitors, thus mediating the development of cervical cancer.

**FIGURE 6:**
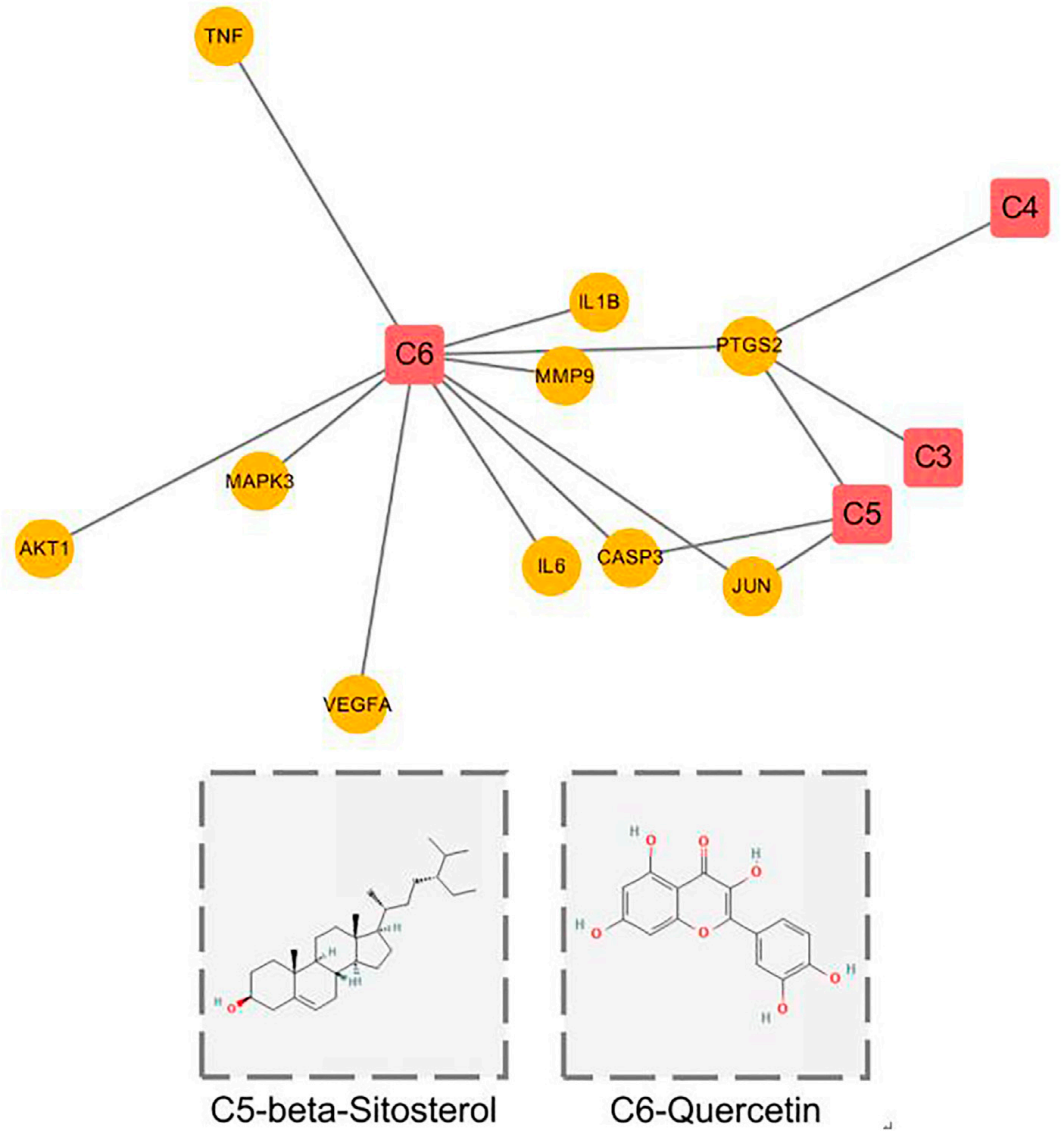
Core target component network and chemical structures of C5 and C6.

In addition, we also constructed the core target-related component network (Figure 6). Only four components (C3, C4, C5, C6) were found to act on the core targets, among which C5(beta-Sitosterol) and C6(Quercetin) acted on the targets with poor prognosis. In addition, whether in the previous component-target network (Figure 1), or in the core target component network (Figure 6). Compared with other components, both C5 and C6 have larger degree value and more targets, suggesting that these two components play a leading role in the whole network. The above analysis results show that various active ingredients contained in Hedyotis diffusa interfere with the progress of cervical cancer by acting on its target network to mediate various pathways, thus exerting curative effects. Among them, beta-Sitosterol and Quercetin may play a key role, and the analysis results need further verification by subsequent experiments.

## Discussion

**FIGURE 7:**
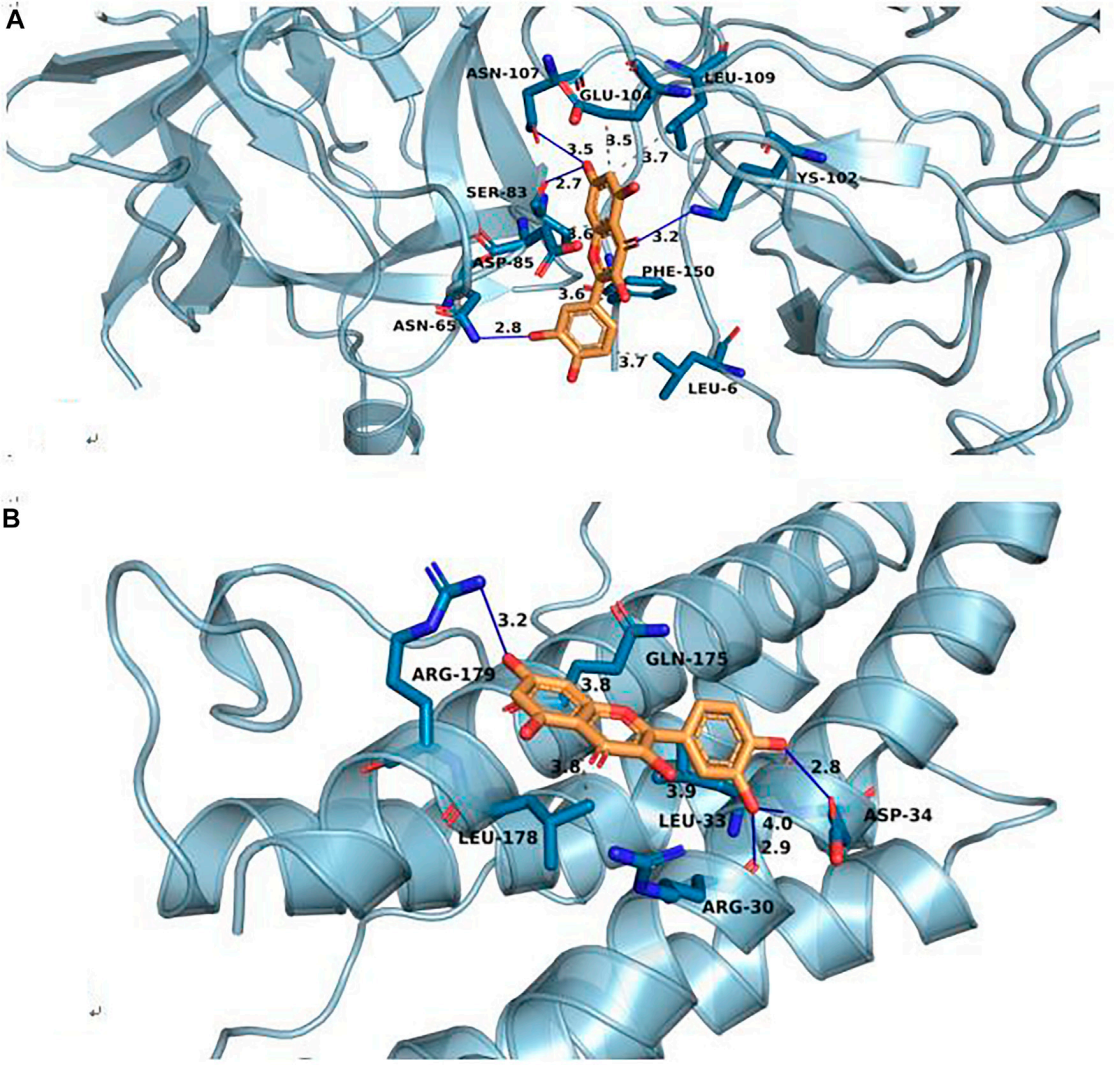
Molecular docking of IL1B-Quercetin **(A)** and IL6_Quercetin **(B)**.

Cervical cancer is a common gynecological malignant tumor, with the mortality rate second only to that of ovarian cancer, and it is one of the malignant tumors threatening the health of women. Western medicine currently mainly adopts radiotherapy, chemotherapy and surgery, while radiotherapy and chemotherapy tolerance has become one of the main causes for the treatment failure, cancer metastasis and recurrence of cervical cancer (Vänskä et al., 2021). In recent years, Chinese medicine has been widely used in clinical due to its advantages of multi-channel, multi-target and small adverse reactions. Modern studies have shown that Hedyotis diffusa has the effects of anti-tumor, anti-oxidation, anti-inflammation and enhance the body’s non-specific immune function. Hedyotis diffusa is often used to treat various tumors in clinic, especially gynecological tumors like cervical cancer, and has obtained good clinical efficacy. However its mechanism of action in the treatment of cervical cancer has not yet been elucidated. Based on the multi-component, multi-target, multi-channel research ideas, this work elucidated the potential mechanism of Hedyotis diffusa in the treatment of cervical cancer from the microscopic point of view by means of network pharmacology. In this study, six important active components related to cervical cancer were screened from Hedyotis diffusa Willd, which correspond to 175 targets of cervical cancer. The six important active ingredients in Hedyotis diffusa for treating cervical cancer were beta-Sitosterol,Quercetin, Poriferasterol,Stigmasterol, 2-methoxy-3-methyl-9,10-anthraquinone and (4aS,6aR,6aS,6bR,8aR,10R,12aR,14bS)-10-hydroxy-2,2,6a,6b,9,9,12a-heptamethyl-1,3,4,5,6,6a,7,8,8a,10,11,12,13,14b-tetradecahydropicene-4a-carboxylic acid, of which the first two components played a more important role in the treatment of cervical cancer than other components. Quercetin is a kind of flavonoid, which widely exists in many fruits and vegetables in nature, and has many pharmacological activities such as anti-tumor, anti-virus, anti-inflammation, anti-oxidation and so on (Gibellini et al., 2011). Madhumitha Kedhari Sundaram et al. studied the mechanism of quercetin in the treatment of cervical cancer through relevant experiments. The results showed that quercetin could induce DNA damage, reduce the activity of cancer cells, and promote the apoptosis of cancer cells by promoting G2-M cell cycle arrest. In addition, quercetin can also block PI3K and other signaling pathways and inhibit the activity of anti-apoptotic proteins to promote cancer cell apoptosis (Darband et al., 2020). Related studies have shown that beta-Sitosterol has curative effect on a variety of cancers, such as cervical cancer, breast cancer, colon cancer, prostate cancer, and so on (Choudhary and Tran, 2011). Studies by Dali Cheng et al. have shown that beta-Sitosterol can inhibit the proliferation of cervical cancer cells and promote the apoptosis of cervical cancer cells. which is mainly related to the expression of P53 and HPVE six proteins (Cheng et al., 2015). The core targets of Hedyotis diffusa in treating cervical cancer mainly included AKT1, VEGFA, TNF, IL6, PTGS2, JUN, IL1B, CASP3, MAPK3, and MMP9. In addition, by analyzing the survival prognosis of the core targets, we found that the four genes with poor prognosis were namely VEGFA, IL1B, IL6 and JUN. VEGFA, as a member of VEGF family, is the most potential signaling protein to promote angiogenesis (Bagri et al., 2010). Baohuan Chen et al. found that VEGFA may inhibit the proliferation and metastasis of cervical cancer cells through Akt/mTor/PI3k signaling pathway (Chen et al., 2014). IL6, a multifunctional cytokine, plays an important role in regulating the growth of tumor cells (Kishimoto, 1989). Lin-Hung Wei et al. showed that IL-6 can regulate the expression of apoptosis protein Mcl-1 in cervical cancer cells and promote the occurrence of cervical cancer (Wei et al., 2001). Recent studies have shown that mi R-125 can inhibit the progression of cervical cancer by inhibiting the VEGF and PI3K-Akt signaling pathways (Fu et al., 2020). IL1B, a pro-inflammatory cytokine, is related to tumorigenesis. Studies have shown that the level of IL1B in plasma of patients with cervical cancer was significantly higher than that of the control group, and the expression level of IL1B played an important role in the carcinogenesis of cervical cancer (Qian et al., 2010). In order to further verify our screening results, we selected C6- quercetin, an important active compound in the treatment of cervical cancer by Hedyotis diffusa, for molecular docking with the core targets IL1B and IL6 which have prognostic value for cervical cancer respectively. Generally, when the binding energy is less than 0, the compound and protein can spontaneously combine, and the lower the binding energy, the greater the possibility of interaction between the compound and protein. Docking score ≤ −5.0 kJmol−1 indicates that it has good binding activity. The molecular docking results are shown in Figure 7, IL1B-quercetin (−8.2) and IL6-quercetin (−7.1). The results show that the core component C6- quercetin predicted in this study has strong binding ability with key targets (IL1B and IL6) and the binding ability of C6- quercetin with target IL1B is stronger, which further confirms the reliability of the prediction results of network pharmacology. The main signal pathways involved in the treatment of cervical cancer by Hedyotis diffusa were pathways in cancer, hepatitis B and blader C. Pathways in cancer, Hepatitis B, Bladder cancer, Prostate cancer, Pancreatic cancer, TNF signaling pathway, HIF-1 signaling pathway, Non-small cell lung cancer, Chagas disease (American trypanosomiasis), Leishmaniasis and so on.

Through the above analysis, we finally screened the active product capable of interfering with the progression of cervical cancer from Herba Hedyotis, and initially explored the multi-pathway action mechanism of Herba Hedyotis in the treatment of cervical cancer by the analysis method of network pharmacology, which provided reference value and theoretical basis for future clinical research. The experimental research method is reasonable, but the potential mechanism of *Melastoma dodecandrum* against cervical cancer is not yet clear, and further experiments are needed.

## Data Availability

The original contributions presented in the study are included in the article/Supplementary Material, further inquiries can be directed to the corresponding authors.
